# A Person-Centred Approach to Communicating Risk

**DOI:** 10.1371/journal.pmed.0020041

**Published:** 2005-02-22

**Authors:** Andy Alaszewski

## Abstract

Standard approaches to communicating risk to patients do not appear to be very effective, argues Alaszewski. We need a new approach that takes patients' own perceptions into account

Doctors and other health professionals play a key role in communicating risk information. They are advisers to patients, especially when patients have to make fateful decisions that can irrevocably change their lives. There is a developing body of literature on the ways in which risk information can be effectively communicated [1,2]. However, much of this literature focuses on the nature of risk information and ways in which the transfer of this information can be improved. It does not fully take into account the complexity of the real world of clinical practice, nor the importance of considering patients as active partners in communication.

## The Rational Model of Risk Communication

Much of the discussion of risk communication is grounded in the rational model of risk communication [3,4]. This model emphasises the role and position of experts such as doctors who have the ability to identify relevant risk knowledge. In the context of medical decision-making this is knowledge about the probable consequences of different courses of action based on scientific research. The role of the doctor is to make such knowledge available so that the patient can then use it to make an informed decision.

With the rational model, when there is evidence that patients have *not* used risk knowledge effectively, then the response of the professional is to consider ways in which risk communication can be improved, such as by improving its presentation or mode of communication. When patients appear to be making irrational or harmful decisions, for example, continuing to smoke, choosing not to vaccinate a child against measles, mumps, and rubella, or not complying with medication, the professional's response is to work harder to convey the risks.[Fig pmed-0020041-g001]

**Figure pmed-0020041-g001:**
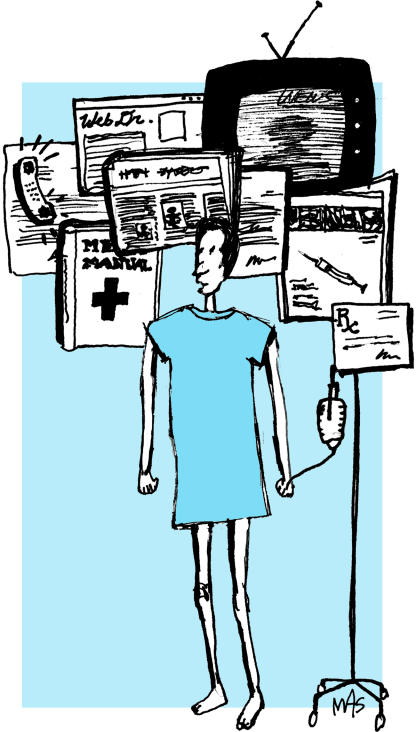
Patients actively seek information on risks from many different sources (Illustration: Margaret Shear, Public Library of Science)

But the rational model contains two key flaws. One relates to the nature of risk knowledge and the second to the nature of communication [5]. Within the rational model, risk knowledge is treated as a relatively simple and straightforward matter—in other words, there is a single uncontested source of knowledge that is relatively easy to access. In reality, risk knowledge is often a complex matter. While such knowledge may be produced by scientific research, it can and often is contested. There may be a scientific consensus, for example, that eating beef or having your child vaccinated against measles, mumps, and rubella is relatively safe, but there are often alternative scientific views, sometimes represented by high-profile media “mavericks” who emphasise the potential hazards [6].

Risk knowledge cannot actually be used directly by patients to inform their decision-making. Scientific research such as in epidemiology generates knowledge about the probability of harmful events occurring within *populations*. Individual patients need information on their own *personal* risks. Expert assessments of risk tend to focus on the knowable and measurable components of risk, that is, the extent to which future events are the same as and predictable by the knowledge of past events. Such assessments by definition exclude uncertainty—those aspects that cannot be assessed and measured. Given the speed of social and technological change, it is not clear that the past is an effective guide to the future. As such, there is an increasing awareness of the uncertainty of risk assessment, for example, in relationship to new diseases such as HIV/AIDS or new technologies such as mobile phones or genetically modified foods.

## The Need for a Person-Centred Approach

Within the rational model of risk communication, the emphasis is on the flow of knowledge from the knowledgeable doctor to the uninformed patient. However, communication is a two-way process, and increasingly there is awareness of the active role of patients and the public [7]. Patients actively seek information, especially when they are aware that they are facing a crucial decision. While they can use traditional sources such as friends and relatives, if they have the skills and resources they can, through media such as the Internet, access highly sophisticated risk knowledge. For example, via the Cochrane Collaboration Web site (www.cochrane.org) they can find the latest evidence-based assessments of medical treatments and technologies, or via the Dr. Foster Web site (www.drfoster.co.uk) they can find the risks associated with different treatment facilities in the United Kingdom. Many patients access a variety of different sources, so they can clearly compare and evaluate the information provided by each.

Patients do give particular credibility to sources that they know, which may include family and friends but also medical advisers with whom they have developed a relationship. They are particularly concerned about the trustworthiness of particular sources. While individuals can use their personal experience to evaluate the trustworthiness of personal sources, such as a particular relative or doctor, they often use contextual information to judge the trustworthiness of impersonal sources [8]. For example, information provided by a source that has an identifiable commercial interest, such as a company marketing a food product, will be considered as less trustworthy than a source without such an interest, for example, an expert committee of scientists.

Patients will actively interpret risk information. If the information is timely and relevant it will tend to be accepted. Patients tend to define relevance in terms of the way they view or frame a situation, and there may be considerable differences between the ways that experts and patients view the same situation. As Zinn notes, the ways in which individuals frame and perceive risk will be highly influenced by their social situation, especially their personal biography [9]. Individuals may identify and respond to the same risks in very different ways. For example, Ziegeler has shown how the background and social context of individuals who have been diagnosed as having multiple sclerosis influence the ways in which they identify and manage their risks and opportunities [9].

## Features of a Person-Centred Approach

Standard approaches to risk communication, whether targeted at groups or individuals, do not appear to be very effective. For example Ruston and Clayton have shown the ways in which women disregard information and conceptually distance themselves from the risk of coronary heart disease—this applies even to those admitted to hospital with the disease [10]. Coleman has documented the failure of strategies that focus on providing information about the risks of teenage pregnancy to have any marked effect [11]. If doctors want to communicate effectively, then they need to develop a person-centred approach to risk communication, one that recognises that communication forms part of a relationship and builds upon it. Communication should be a dialogue that develops as the relationship develops, and those involved should have complementary and linked roles.

Thus, the initial stage of communication could involve identifying the key issues, that is, those that cause concern for the patient. In this phase the emphasis might be on the patient talking and the doctor listening. If there is a major difference in the ways in which patient and doctor are framing the risk issues, then there might need to be an exchange or negotiation in which both parties adjust their mutual expectations and seek a mutually acceptable definition of what the problem is. If such an exchange does not take place, and if the patient's definitions are disregarded and not acknowledged, there is the danger that the patient will passively acquiesce but treat much of the information provided by the doctor as irrelevant and disregard it.

If and when there is agreement, then there is the possibility of discussing the future and the likely consequences of taking different actions (risk communication in its traditional form). During this part of the exchange the emphasis might shift towards the doctor talking more and the patient listening more. There is a transfer of information, but it is a two-way process. The doctor should learn something about the patient's situation, including the risks that the patient is concerned about and the patient's beliefs about the nature of such risks. The patient should learn about the doctor's views of the nature of the risks that the patient is facing and the options for managing such risks.

Underpinning the development of an effective relationship is the development of mutual trust. While trust usually takes time to develop, it is possible even during a short but positive exchange, in which mutual respect is shown, for a form of “swift” trust to be developed [12].

While I have focussed on communication in face-to-face relationship, the same issues and processes can be identified in more impersonal communication, such as the provision of risk information in health promotion campaigns. In such campaigns special mechanisms need to be created for dialogue. For example, Jones has described a project that engages young drug users in Hong Kong by helping them make videos about drug use, and has shown how such techniques can be used to evaluate and improve current health promotion adverts [13].

## Conclusion

There are no quick technical fixes for communicating risk information. If health professionals are serious about communicating risk information so that patients and others can make informed choices, they need to recognise that communication is a two-way process, and they need to take time to access patients' accounts and perceptions. Such investment should pay off both in an improved relationship and also in improved concordance with treatments.
